# The effects of chromium propionate supplementation to yearling steers in a commercial feedyard on growth performance, carcass characteristics, and health

**DOI:** 10.1093/tas/txad078

**Published:** 2023-07-18

**Authors:** Sara J Trojan, Jerilyn E Hergenreder, Landon G Canterbury, John T Leonhard, William D Clark, Jonathan L Beckett, John M Long

**Affiliations:** Peak Beef Nutrition and Management Consulting, LLC, Casper, WY 82604, USA; Kemin Industries, Inc., Des Moines, IA 50317, USA; Kemin Industries, Inc., Des Moines, IA 50317, USA; Kemin Industries, Inc., Des Moines, IA 50317, USA; Kemin Industries, Inc., Des Moines, IA 50317, USA; Beckett Consulting Services, Ft. Collins, CO 80524, USA; Deseret Cattle Feeders, LLC, Satanta, KS 67870, USA

**Keywords:** carcass, chromium propionate, growth, health, yearling steers

## Abstract

British crossbred steers (*n = *3,072; initial body weight [**BW**] = 358 ± 37 kg) were used to evaluate the effects of chromium propionate supplementation to yearling steers in a commercial feedyard on growth performance, carcass characteristics, and health. Steers were blocked by initial BW; pens were assigned randomly to one of two dietary treatments within block. Treatments, replicated in 15 pens per treatment with 75 to 135 heads per pen, included 1) control, 0 mg supplemental Cr/kg dietary dry matter (**DM**) (**CTL**); 2) 0.50 mg supplemental Cr/kg diet DM (chromium propionate; KemTRACE Chromium 0.4%, Kemin Industries, Des Moines, IA) (chromium propionate, CR). Final BW (638 vs. 641 kg), average daily gain (1.81 vs. 1.82 kg), DM intake (11.02 vs. 11.02 kg), and gain efficiency (0.164 vs. 0.165) did not differ between CTL and CR, respectively (*P *≥ 0.75). No differences among treatments for hot carcass weight (407 vs. 408 kg, CTL and CR, respectively), dressing percentage, longissimus muscle area, or yield grade were observed (*P* ≥ 0.15). Twelfth-rib fat thickness tended (*P* = 0.10) to be greater for CR vs. CTL (1.55 vs. 1.29 cm, respectively). A trend (*P* = 0.10) for marbling score to be higher for CR vs. CTL was detected (452 vs. 440, respectively). Distribution of quality grade was similar between CR and CTL; 1.52% of carcasses graded prime (*P *= 0.68), and 87.2% of carcasses graded choice (*P* = 0.68). Respiratory morbidity was low (1.93%) and not different among treatments (*P *= 0.20); likewise, there was no difference in respiratory treatment rates between treatments (*P ≥ *0.18). Supplementing Cr to high-performing yearling steers did not alter growth performance, carcass characteristics, or health outcomes.

## Introduction

Chromium is a trace element and essential nutrient that plays a vital role in glucose metabolism by potentiating insulin-sensitive glucose uptake (Schwarz and Mertz, 1959; Vincent, 2000). In 2009, the US FDA permitted the inclusion of Cr propionate in diets for cattle up to 0.50 mg/kg dietary dry matter (**DM**) (Kemin Industries, Inc., Des Moines, IA); Cr propionate remains the only organic Cr source approved for cattle in the US.

A multitude of studies have been conducted to better understand the mode of action, feeding application, and biological response to supplemental Cr in cattle. In general, data for feedlot growth and carcass performance are inconsistent; the feeding rate and duration vary, and the response to long-term Cr supplementation among cattle of different genotypes and backgrounds is poorly understood. In beef cattle, Cr propionate improved the efficiency of glucose metabolism (Spears et al., 2012), enhanced immune function, and reduced morbidity in high-risk feedlot cattle during the receiving phase (Burdick et al., 2011; Bernhard et al., 2012a; Smock et al., 2020a). Yet, responses to Cr supplementation on feedlot growth and carcass performance are variable and influenced by disparities in Cr source, feeding level and duration, and cattle physiology. Chromium supplementation improved feedlot performance (Barajas et al., 2008; Baggerman et al., 2020; Hallmark et al., 2020) and increased hot carcass weight (Budde et al., 2019; Baggerman et al., 2020; Hallmark et al., 2020); whereas others have reported no difference in feedlot growth (Kneesken et al., 2016) or carcass performance for Cr supplemented cattle (Kneesken et al., 2016; Van-Bibber Kruger et at. 2016).

Research with Cr propionate fed to cattle for the duration of the feeding period is limited. Previous work with Cr has been conducted on an experimental basis with substantially fewer animals per pen than in a practical commercial feedlot setting, and at an inclusion level that is less than the maximum approved feeding level. The objective of this experiment was to evaluate the effects of feeding Cr propionate at 0.50 mg/kg of dietary DM throughout the feeding period to British cross-yearling steers on growth performance, carcass characteristics, and health in a large-pen commercial feedyard setting. Our hypothesis was that Cr supplemented at 0.50 mg/kg of dietary DM would improve carcass performance and decrease respiratory morbidity.

## Methods

Study procedures were performed in accordance with the guidelines by the Federation of Animal Science Societies (2020) for the management and use of beef cattle in research.

### Cattle Management and Treatments

British crossbred yearling steers (*n* = 3,072) were received at a feedyard in Southwest, KS on June 15, 17, and 23, 2020. Steers grazed native and improved pastures surrounding the feedyard for 86 d, from weaning to feedyard pen placement. Within 24 h of feedyard pen placement, cattle were individually identified with duplicate ear tags with a unique individual animal number and a solid-colored identification tag. Cattle were vaccinated for infectious bovine rhinotracheitis, and bovine viral diarrhea virus types 1 and 2 (Titanium 3; Elanco Animal Health, Greenfield, IN); treated for external parasites with Dectomax (Doramectin, Zoetis, Kalamazoo, MI) and internal parasites with Safe-Guard (Safe-Fenbendazole, Intervet, Inc., Millsboro, DE, Boehringer Ingelheim). All steers were implanted with Revalor-XS (200 mg Trenbolone Aetate and 40 mg Estradiol, Merck Animal Health, Summit, NJ). Cattle enrolled in the study were deemed visually healthy by trained personnel; cattle with unmanageable temperaments, breed anomalies, injuries, or body weight (**BW**) deviating significantly from the mean were omitted from the study.

Following arrival processing, cattle were fed a common receiving ration for 1 to 2 d and then weighed individually in a hydraulic chute (Bowman Manufacturing, Fremont, NE) on load cells (Bowman Manufacturing) with weights indicated by Digi-Star SW600 scale head (Fort Atkinson, WI); individual scale readability was 2.25 kg. The scale was certified every two years and verified up to 90.7 kg prior to daily use. Enrolled steers were ranked by ascending BW, assigned to block by weight and arrival date (*n *= 3,072), steers within block were assigned randomly to pens (75 to 135 steers/pen), and pens within block were assigned randomly to 1 of 2 dietary treatments within block; thus, treatments were replicated in 15 pens. Prior to study initiation, pen weights were obtained to ensure pen weight uniformity. Steers were allocated into 30 contiguous, open air, soil-surface pens with a minimum of 13.9 m^2^/animal and a minimum of 30.48 cm of linear bunk space. Treatments, arranged in a randomized complete block design included: 1) control, 0 mg supplemental Cr/kg dietary DM (**CON**); 2) 0.50 mg supplemental Cr/kg dietary DM (Cr propionate; KemTRACE Chromium 0.4%, Kemin Industries, Des Moines, IA; CR). Chromium propionate was added to the basal diet through a micro-ingredient machine maintained by Animal Health International (Spanish Fork, Utah). All cattle received a steam-flaked/high moisture corn-based diet, including a vitamin-trace mineral supplement to meet or exceed NASEM (2016) recommendations. Feed additives, including an ionophore, antimicrobial (liver abscess prevention), and ractopamine-hydrochloride, administered the final 30 d on feed (Optaflexx, Elanco Animal Health), were also delivered through the micro-ingredient machine. Ingredient and analyzed nutrient composition of the finishing ration is shown in Table 1. Cattle were on feed for 156 d.

**Table 1.** Ingredient and nutrient composition of the finishing diet

	Treatment^1^	
	CON	CR
Ingredient, %		
Steam flaked corn	63	63
High moisture corn	20	20
Dried distillers grains	6	6
Liquid supplement	4	4
Ether extract	2.7	2.7
Alfalfa hay	2	2
Triticale silage	2	2
Micro-ingredients^2^	0.3	0.3
KemTRACE chromium 0.4%^3^	—	
Composition, % of dry matter		
Net energy, maintenance, Mcal/kg^4^	2.22	2.22
Net energy, gain, Mcal/kg^4^	1.54	1.54
Crude protein^5^	14.80	15.00
Calcium^5^	0.59	0.68
Phosphorus^5^	0.46	0.48

^1^CON, control; CR = 500 ppb DM chromium propionate (KemTRACE Chromium 0.4%, Kemin Industries, Des Moines, IA).

^2^Included minerals, vitamins, monensin (Rumensin 90, Elanco Animal Health, Greenfield, IN) to provide 25 mg/kg of ration dry matter and tylosin (Tylan 100, Elanco Animal Health) to provide 11 mg/kg of ration dry matter. For the final 30 d on feed, included ractopamine-hydrochloride (Optaflexx, Elanco Animal Health) to provide 300 mg/steer/d.

^3^KemTRACE Chromium 0.4% (Kemin Industries, Des Moines, IA) included in the micro-ingredient machine to supply 500 ppb DM chromium propionate in the CR diet.

^4^Formulated values based on NASEM (2016).

^5^Analysis by Servi-Tech Laboratories, Dodge City, KS.

Steers within respective weight blocks were transported 101.5 km to a commercial abattoir (Cargill Meat Solutions, Dodge City, KS) on October 22, 30, November 06, 11, 17, and December 11, 18, and 21, 2020. For each slaughter date, steers were shipped in the morning, with pen weight obtained prior to shipping. Final pen weights were shrunk 4% to determine final BW.

### Feeding and Health Management

Steers were acclimated to a 90% concentrated diet with transition diets. Throughout the study duration, feed bunks were read twice daily between 0700 and 0800 hours and between 1400 to 1600 hours; unconsumed feed was documented. Daily feeding amounts were determined by the consumption observations of the previous day. Diets were mixed daily in a Roto-Mixer, and mixing times were standardized for each treatment with electronic timers. Feed truck cleanout occurred daily between treatment deliveries to ensure no cross-contamination of diets. Feed samples were collected daily from each ration to determine daily DM. Diets were analyzed for Cr prior to study initiation; however, analyzed dietary Cr levels were determined to be of low biological value as Cr contamination of feedstuffs by soil and metal contributes significantly to the analyzed Cr content (Spears et al., 2017).

Health management was in accordance with the commercial feedyard standard operating procedures.

### Carcass Characteristics

Carcass characteristics were evaluated 24 h after slaughter. All carcass measurements were captured by packing plant camera data and verified by USDA personnel for quality grade and yield grade. Dressing percentage was calculated by dividing the hot carcass weight by the shrunk final BW.

### Statistical Analysis

Data for growth performance and carcass characteristics were analyzed using the MIXED procedure of SAS (SAS Inst. Inc., Cary, NC). Pen was the experimental unit, treatment was a fixed effect, and block was a random effect, with the Kenward-Roger adjustment included for degrees of freedom. Binomial proportions were used to analyze the distributions of quality grade, yield grade, morbidity, mortality, and incidence of digestive conditions using the GLIMMIX procedure of SAS, block was included as a random effect. The protected *F*-test was applied, and when significant, least squares means were separated and reported using the LSD procedure of SAS (α = 0.05). *P-*values between 0.05 and 0.10 were discussed as trends.

## Results

There was no difference in final BW, average daily gain (1.81 kg/steer/day), DM intake (11.02 kg), or gain efficiency (0.16) between treatments (*P *≥ 0.75; Table 2). Carcass weight, dressing percentage, longissimus muscle area, percentage kidney, pelvic, and heart fat, and yield grade were not different between treatments (*P *≥ 0.15; Table 3), 12th-rib fat tended (*P* = 0.10) to be greater for CR than CON. The quality grade of the cattle was exceptional; 88.6% of carcasses graded choice or better and was not influenced by treatment (*P = *0.68; Table 3).

**Table 2.** The effect of chromium propionate supplementation on growth performance of yearling feedlot steers

Item	Treatment^1^		SEM^2^	*P-*Value
	CON	CR		
Days on feed	156	156		
Initial body weight, kg	357	359	9.35	0.92
Final body weight, kg	639	641	7.88	0.75
Average daily gain, kg	1.81	1.82	0.05	0.93
Dry matter intake, kg	11.02	11.02	0.19	0.98
Gain:feed	0.1647	0.1650	0.0017	0.87

^1^CON, control; CR = 0.50 mg supplemental chromium propionate/kg diet DM (KemTRACE Chromium 0.4%, Kemin Industries, Des Moines, IA).

^2^Standard error of least square mean.

**Table 3.** The effect of chromium propionate supplementation on carcass characteristics of yearling feedlot steers

Item	Treatment^1^		SEM^2^	*P-*Value
	CON	CR		
Hot carcass weight, kg	407	408	5.87	0.84
Dressing percent	63.7	63.6	0.21	0.67
12^th^-rib fat, cm	1.52	1.56	0.06	0.10
Longissimus muscle area, cm^2^	96.81	95.90	1.12	0.55
Kidney, pelvic, heart fat, %	3.33	3.33	0.06	0.97
Calculated yield grade	2.61	2.71	0.06	0.15
Marbling score	465	467	8.92	0.78
Quality grade				
Prime, %	1.42	1.62	0.33	0.68
Choice, %	87.40	87.00	0.08	0.68
Select, %	11.21	11.52	0.09	0.74

^1^CON, control; CR = 0.50 mg supplemental chromium propionate/kg diet DM (KemTRACE Chromium 0.4%, Kemin Industries, Des Moines, IA).

^2^Standard error of least square mean.

Overall respiratory morbidity was very low (1.93%; Table 4) and not different among treatments. There was no difference in respiratory retreatment rates (*P *= 0.23), and overall mortality rate was similar among treatments (1.13%; *P* = 0.25).

**Table 4.** The effect of chromium propionate supplementation on health parameters of yearling feedlot steers

Item	Treatment^1^		SEM^2^	*P-*Value
	CON	CR		
Respiratory Morbidity, %	1.60	2.26	0.003	0.20
Temperature, 1^st^ treatment, °C	39.9	38.7	1.20	0.42
Days to 1^st^ treatment	34	33	14.1	0.42
Digestive, %	0.13	0.39	0.001	0.29
Buller, %	0.70	0.71	0.002	0.98
Lame, %	0.13	0.19	0.001	0.66
Railer, %	0.26	0.13	0.001	0.48
Mortality, %	0.90	1.36	0.003	0.25
Respiratory, %	77.78	61.11	0.137	0.45
Digestive, %	22.22	33.33	0.134	0.59

^1^CON, control; CR = 0.50 mg supplemental chromium propionate/kg diet DM (KemTRACE Chromium 0.4%, Kemin Industries, Des Moines, IA).

^2^Standard error of least square mean.

## Discussion

The cattle enrolled in this study were low-risk, evidenced by the low rates of morbidity and mortality, and arrived at the feedyard from adjacent pastures. Growth rates and carcass performance of cattle in this study were excellent.

In general, research with Cr supplementation to feedlot cattle is inconsistent by Cr source and inclusion level. Long-duration feeding of Cr propionate, with a range of Cr inclusion rates (0.25 mg Cr/kg DM to 0.45 mg Cr/kg DM), demonstrated increased live weight gain (Barajas et al., 2008; Baggerman et al. 2020; Hallmark et al., 2020) and carcass weight (Barajas et al., 2008; Budde et al., 2019; Baggerman et al., 2020; Hallmark et al., 2020). Yet, Cr propionate fed in combination with yeast products, providing 3.2 mg Cr/steer daily did not show a difference from control for feedlot growth or carcass characteristics (Van-Bibber Kruger et al., 2016). Sánchez -Mendoza et al., 2014 supplemented chelated Cr combined with yeast products, providing 500 ppb/head daily in a high ambient temperature area and did not report a difference for feedlot growth performance but indicated that the Cr product increased longissimus muscle area and tended to improve retail yield. Steers (*n* = 17 per treatment) supplied 0.30 mg Cr/kg DM from Cr propionate had similar feedlot growth performance and carcass variables to control (Kneeskern et al., 2016). Supplementing 3 mg/steer daily Cr propionate during the beta-agonist period, at the end of the feeding period, for a short duration only, did not result in a difference from control for feedlot growth or carcass parameters (Bohrer et al., 2014; Edenburn et al., 2016).

In a survey to consulting feedlot nutritionists, Samuelson et al. (2016) reported that all surveyed nutritionists fed trace minerals at rates numerically greater than the NASEM (2016) requirement. Niedermayer et al. (2018) compared supplementing trace minerals to feedlot diets at NASEM (2016) requirements with feeding rates by independent nutritionists and indicated that alterations of trace mineral feeding rates, beyond the requirement may be necessary for optimal feedlot growth performance. Further, genetic differences of modern cattle types and enhanced growth promotion technologies may alter trace mineral requirements (Genther-Schroeder et al., 2016). Understanding how trace minerals interact is important for optimizing performance. Two recent studies have evaluated the interactive effects of Zn and Cr propionate during the finishing period. Supplementing 90 mg Zn/kg DM and 0.25 mg Cr/kg DM increased average daily gain 0.07 kg and hot carcass weight 8.5 kg compared to steers-fed diets without supplemental Cr (Budde et al., 2019). Similar results were reported by Hallmark et al. (2020) such that average daily gain was greater and hot carcass weight tended to be heavier for steers supplied 30 or 90 mg Zn/kg DM with 0.25 mg Cr/kg DM than steers only supplemented Zn, regardless of level of supplemental Zn.

Although there is not presently a Cr requirement for beef cattle, Cr deficiencies are revealed during stress. Chromium excretion increases during acute and chronic stress (Anderson et al., 1991; Anderson, 2003). Cattle background, management, weather, and overall metabolic load are variable in cattle feeding and influence the degree of stress that cattle endure throughout the feeding period. During a 56-d receiving period, high-risk steers supplemented 0.30 mg/kg dietary DM Cr propionate had higher average daily gain, improved gain efficiency, and 18.4% less first-treat respiratory morbidities than unsupplemented steers (Bernhard et al., 2012b). During a lipopolysaccharide challenge, supplementing 0.20 mg/kg of dietary DM Cr propionate improved immune response parameters (Burdick et al., 2011). Smock et al. (2020a, 2020b) reported a reduction in first-treat respiratory morbidity (43.9% vs. 35.8%, control or Cr, respectively), and improved immune function by supplementing 0.30 mg/kg dietary DM Cr propionate. In high-stress cattle populations, Cr supplementation with a high-Cr yeast product decreased serum cortisol, increased total immunoglobulin production (Chang and Mowat, 1992), and decreased morbidity (Moonsie-Shageer and Mowat, 1993). Yet, when no morbidity was observed during a 56-d study, supplementing 0.4 mg/kg DM of variable Cr sources (Cr chloride or Cr-nicotinic acid) did not impact growth performance (Kegley et al., 1997). The positive responses to Cr supplementation on immunity are likely attributable to the ability of Cr to potentiate the action of insulin, enhancing glucose uptake, especially when insulin production is altered by glucocorticoid production. Perhaps the overall lack of significant response to Cr supplementation in this experiment is due to the overall low-stress nature of enrolled cattle.

Knowledge of mode of action of Cr in beef cattle has expanded over time. Chromium’s ability to enhance the efficiency of glucose metabolism by augmenting the action of insulin in cattle has been well-documented (Kegley et al., 1997; Stahlhut et al., 2006; Spears et al., 2012). Insulin levels were less, and the insulin:glucose ratio was lower for heifers supplemented with chromium propionate compared with control (Spears et al., 2012). Having less circulating insulin upregulates gluconeogenesis in ruminants as insulin decreases the gluconeogenic pathway by blocking the pancreatic release of glucagon (Greenbaum et al., 1991).

Over 80% of skeletal muscle tissue relies upon insulin-dependent glucose uptake mechanisms (McGilcrest et al. 2011). The role of Cr in protein synthesis was demonstrated by Baggerman et al. (2020); feedlot steers fed 0.45 mg/kg DM Cr propionate had an increased upregulation of glucose transporter type-4 in skeletal muscle tissue compared with control, and a subsequent increase in hot carcass weight. Type-4 glucose transporters are insulin-dependent receptors and are responsible for shuttling extracellular glucose into adipose and muscle tissue (Sano et al., 2003). Although there was not a significant change in carcass weight in the present study, other work has illustrated this response (Barajas et al., 2008; Budde et al., 2019; Hallmark et al., 2020). Further, Cr may affect lipid metabolism as several studies have indicated a reduction in nonesterified fatty acid production with supplementation of Cr from variable sources (Kitchalong et al., 1995; Gentry et al., 1999; Bernhard et al., 2012a), suggesting that Cr supplementation alters the metabolic demand for lipolysis.

The overall lack of response to Cr supplementation in the present study is likely due to the nature of the low-risk study population as the cattle were heavy weight yearlings that experienced minimal management stress upon feedyard arrival.
